# A rare cardiopulmonary parasite of the European badger, *Meles meles*: first description of the larvae, ultrastructure, pathological changes and molecular identification of *Angiostrongylus daskalovi* Janchev & Genov 1988

**DOI:** 10.1186/s13071-016-1718-8

**Published:** 2016-08-02

**Authors:** Călin Mircea Gherman, Georgiana Deak, Ioana Adriana Matei, Angela Monica Ionică, Gianluca D’Amico, Marian Taulescu, Lucian Barbu-Tudoran, Alexandru Sarmaşi, Andrei Daniel Mihalca, Vasile Cozma

**Affiliations:** 1 Department of Parasitology and Parasitic Diseases, Faculty of Veterinary Medicine, University of Agricultural Sciences and Veterinary Medicine Cluj-Napoca, Calea Mănăştur 3-5, 400372 Cluj-Napoca, Romania; 2 Department of Pathology, Faculty of Veterinary Medicine, University of Agricultural Sciences and Veterinary Medicine Cluj-Napoca, Calea Mănăştur 3-5, 400372 Cluj-Napoca, Romania; 3 Department of Molecular Biology and Biotechnology, Faculty of Biology and Geology, Babes-Bolyai University, M. Kogălniceanu Street, 1, Cluj-Napoca, 400648 Romania

**Keywords:** *Angiostrongylus daskalovi*, Badger, *Meles meles*, Romania, SEM, Histopathology, Molecular analysis

## Abstract

**Background:**

*Angiostrongylus daskalovi* is a rare cardiopulmonary nematode infecting badgers. The parasite was described in 1988 and, since then, found only once in mustelids in Europe. The present study aims to report new cases of patent *A. daskalovi* infection in badgers from northern Romania and to provide new information on its ultrastructure, molecular diagnosis, and pathology.

**Methods:**

Eight road-killed or hunted badgers originating from Maramureș and Alba counties in Romania were collected and necropsied. Adults and larvae of cardio-pulmonary nematodes were collected and examined by light and scanning electron microscopy (SEM). Genomic DNA was extracted from adults and first-stage larvae (L1). PCR amplification of the internal transcribed spacer 2 (ITS2, ∼500 bp) of the rRNA gene was performed. Amplicons were purified, sequenced, and compared to those available in the GenBank database. Histopathological examination of the lungs was performed and lesions described.

**Results:**

The necropsy revealed the presence of nematodes in the pulmonary arteries of three animals. All parasites were mature adults and the coproscopic examination showed the presence of eggs and L1 larvae in all three positive animals. Light microscopy examination confirmed the morphological and morphometric similarity of parasites to *Angiostrongylus daskalovi*. SEM highlighted the typical angiostrongylid structure of the rays of the copulatory bursa and the anterior extremity, with the presence of six sensory papillae surrounding the mouth opening in which a triangular tooth was visible. The first-stage larva (L1) of *A. daskalovi* is described here for the first time. Histopathological examination of the lungs showed chronic interstitial verminous pneumonia due to the presence of adult parasites. Molecular analysis showed 100 % nucleotide similarity to an *Angiostrongylus* sp. isolate originating from a badger from Spain, tentatively identified as *A. daskalovi*.

**Conclusions:**

Our study unequivocally demonstrates the presence of *A. daskalovi* in European badgers from Romania, provides the first description of the larvae and reveals new data about the ultrastructure of adult parasites and their pathological impact, contributing to the understanding of the phylogenetic relationships with other congeneric species.

## Background

The family Mustelidae is the richest group within the order Carnivora, comprising five subfamilies: Lutrinae (otters), Melinae (European badgers), Mellivorinae (honey badgers), Taxidiinae (American badgers) and Mustelinae (weasels, tayra, wolverines, martens, polecats) [1]. According to recent multigene phylogenetic analysis, the family is split into four major clades and three monotypic lineages [2]. Among all these mustelids, the European badger (*Meles meles*) is spread throughout Europe and in some parts of the Middle East. It is an opportunistic omnivorous species; its diet includes a broad range of animals and plants. This varied diet exposes the badger to the risk of contamination by a wide variety of cysts, eggs, larvae or intermediate hosts of certain parasites. Of these, cardiopulmonary nematodes represent a particular group, several species being reported in mustelids. The genus *Aelurostrongylus* Cameron, 1927 contains two species that are rarely reported in badgers in Europe: *A. falciformis* Schlegel, 1933 in Italy, Germany, Norway and Great Britain [3–6] and *A. pridhami* Anderson, 1962 in Spain. Two other metastrongyloid species belonging to the genus *Angiostrongylus* Kamensky, 1905 have been recorded in badgers. *Angiostrongylus daskalovi* Janchev & Genov, 1988 was described from the pulmonary arteries of the European badger (*M. meles*) in the north-central region of Bulgaria [7] and more recently in Spain [8]. Additionally, *Angiostrongylus vasorum* (Baillet, 1866) was identified in the cardiopulmonary system of badgers in Switzerland, Italy and Spain [9–12]. Another species, *Angiostrongylus gubernaculatus* Dougherty, 1946, was described from the right ventricle of the Californian badger, *Taxidea taxus neglecta* in California and the California Channel Islands, the United States [13, 14].

Apart from these two genera, other lung nematodes have been reported in European badgers including *Crenosoma* sp., *C. vulpis* (Dujardin, 1845) and *C. melesi* Janchev & Genov, 1988, as well as the trichuroid nematode *Capillaria aerophila* Creplin, 1839 [3, 4, 12, 15, 16]. Cardiopulmonary nematodes have also been reported in other mustelids like the stoat (*Mustela erminea*) and weasel (*Mustela nivalis*) infected with *A. vasorum* [17, 18] and the European pine marten (*Martes martes*) and the beech marten (*Martes foina*) infected with *A. daskalovi* [7].

The European badger is considered the typical host for *A. daskalovi* [7]. This nematode has a poorly known geographical distribution, so far being recorded only in Bulgaria and Spain. Moreover, the life-cycle and the host spectrum are incompletely known; the larvae are unknown and the pathological aspects have never been described. Due to these shortcomings, it is important to add new data regarding the infection caused by this nematode species. In this context, the present paper reports the first cases of patent *A. daskalovi* infection in badgers in Romania, emphasizing the ultrastructure of adult parasites and morphology of the L1 larval stage, molecular characterization, and pathological changes.

## Methods

### Sample origin and collection

**Fig. 1:**
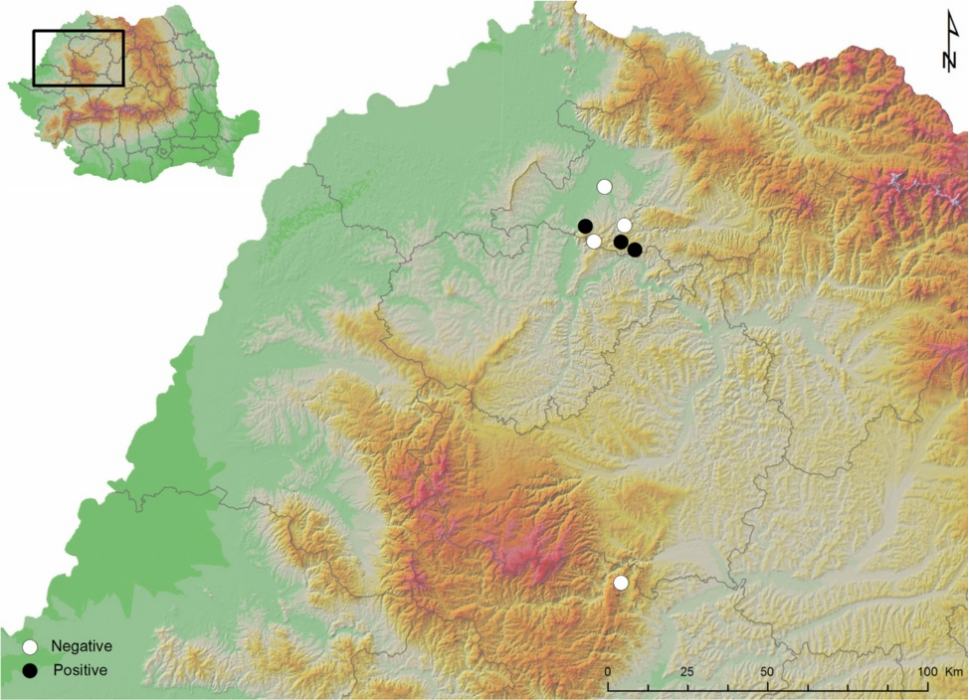
Origin of the samples and positive cases

Between February 2015 and April 2016, eight European badgers (*Meles meles* L.) were collected in the counties of Maramureș and Alba, in northern and central Romania (Fig. 1). The animals were either road-killed or hunted. Their carcasses were submitted for pathological and parasitological examination within a few hours after the death of the animals and examined immediately. During the necropsy, all nematodes found in the pulmonary arteries were collected in formalin (for morphological examination) and absolute ethanol (for molecular analysis). The classical Baermann method [19] was performed on the lung tissue and faeces, and the metastrongyloid first-stage larvae (L1) were collected. The morphology and morphometry under light microscopy of adults and larvae and SEM characteristics of adults were analysed, ten parameters being compared to other reports of angiostrongylid parasites in mustelids (Table 1).

**Table 1:** Morphometric features of *A. daskalovi* and comparative data for other *Angiostrongylus* spp. identified in mustelids

Feature/Species		*A. daskalovi*	*A. daskalovi*	*Angiostrongylus* sp. (badger)	*A. vasorum*	*A. gubernaculatus*
Source		Present study	Janchev & Genov [7]	Gerrikagoitia et al. [8]	Costa et al. [23]	Dougherty [13]
Male		(*n* = 7)				
Body length (mm)		15.8–20.5	13.4–21.3	19.4 ± 7.7	12.4	18.0–19.5
Body width (μm)		253–331	254–306	243 ± 20	243	300
Distance from excretory pore to cephalic end (μm)		227–526	386–463	409 ± 25	373	
Oesophagus length (μm)		312–335	336–366	333 ± 18	220–275	300–355
Spicule length (μm)	shorter	322–352	336–409	346 ± 24	410–485	510–560
	longer	337–380				
Female		(*n* = 16)				
Body length (mm)		24.0–34.0	14.4–31.1	25.0 ± 14.0	15.6	22.0–24.0
Body width (μm)		366–852	340–511	345 ± 18	268	350
Distance from excretory pore to cephalic end (μm)		454–618	379–636	447 ± 102	403	
Oesophagus length (μm)		305–456	356–556	368 ± 17	240–280	335–350
Distance from vulva to anus (μm)		203–515		295 ± 46	141	
Distance from vulva to caudal end (μm)		286–612	269–412	366 ± 44	205	205–250
Distance from anus to caudal end (μm)		60–193	76–115	79 ± 3	67	75–90
Vulvar opening (length/width, μm)		38–40/4–5				
Anus (length/width, μm)		8–10/2–3				

### Pathology

Full necropsy and histological examination were carried out on all badgers included in this study. Selected samples from the right atrium, pulmonary arteries, pulmonary parenchyma and tracheobronchial lymph nodes were collected for histological analysis. Samples were fixed in 10 % phosphate-buffered formalin for 24 h, routinely processed, embedded in paraffin wax, cut into 4 μm sections, and stained with hematoxylin and eosin (H&E).

### Scanning electron and light microscopy

All adult worms were washed in saline, preserved for 24 h in 0.5 % formalin, dehydrated, cleared in lactophenol, mounted in Canada balsam and analyzed by light microscopy using an Olympus BX 61 microscope (Japan). For scanning electron microscopy, some adult parasites were fixed for 2 h at 4 °C in 2.7 % glutaraldehyde in 0.1 M sodium cacodylate buffer (pH 7.2) and washed in PBS. Samples were post-fixed for 1 h with 1 % OsO_4_. The parasites were dehydrated in an ethanol series (30–100 %), and infiltrated with hexamethyldisilazane, dried, mounted on aluminum stubs coated with a 10 nm gold layer, and examined with a Hitachi SU8230 Scanning Electron Microscope (Japan).

### Molecular analyses and species identification

Genomic DNA was extracted from three adult nematodes (one from each positive animal) and 30 L1 stages (a pool of ten from each positive animal) using a commercial kit (Isolate II Genomic DNA Kit, Bioline, London, UK) as stated in the manufacturer’s instructions. PCR amplification of the internal transcribed spacer 2 (ITS2, ∼500 bp) of the rRNA gene was performed using the NC1/NC2 primer pair as previously described [20]. Amplicons were purified using silica membrane spin columns (QIAquick PCR Purification Kit, Qiagen, Halden, Germany) and externally sequenced by Macrogen Europe (Amsterdam). Sequences were compared to those available in the GenBank database by Basic Local Alignment Search Tool (BLAST) analysis. Phylogenetic analyses were conducted using MEGA6 software [21]. The evolutionary history was inferred by using the Maximum Likelihood method based on the Tamura-Nei model [22]. Species identification was based on morphological characteristics, associated with molecular analysis [7, 8].

## Results

### Morphology and morphometry of *Angiostrongylus daskalovi*

**Fig. 2:**
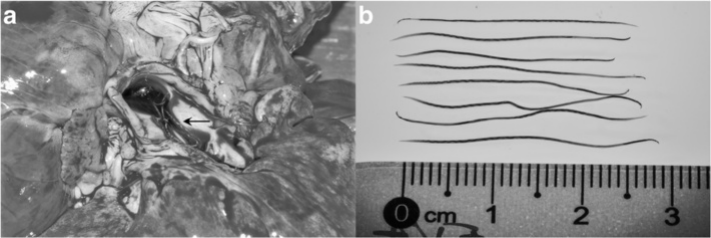
**a** Location of *Angiostrongylus daskalovi* in the pulmonary arteries (*arrow*). **b** Size of *A. daskalovi* females

Seven males and 16 females were collected from the pulmonary vessels of the infected badgers (Fig. 2a). Of these, 9 specimens (3 males and 6 females) originated from the first infected badger, two females from the second and 12 (4 males and 8 females) were collected from the third animal. Adult worms exhibited a pronounced sexual dimorphism, females being larger than males, see Table 1 for detailed morphometric data for the adult worms.

**Fig. 3:**
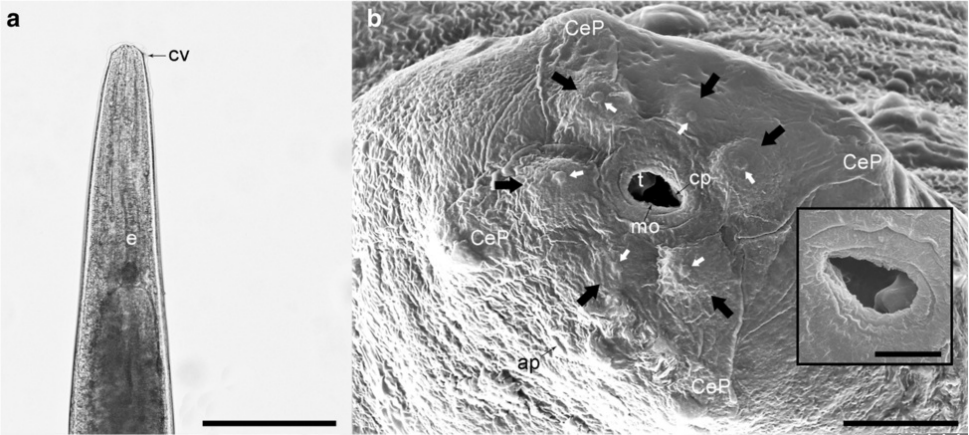
Light microscopy (**a**) and SEM photomicrographs (**b**) of the anterior extremity of *A. daskalovi*: The tooth and the cutting plate are clearly visible in the inset. Six labial papillae (*black arrows*) and six small protuberances (*white arrows*) are indicated. *Abbreviations*: ap, amphidial pore; CeP, four cephalic papillae; cp, cutting plate; cv, small cephalic vesicle; e, oesophagus; mo, mouth opening; t, tooth. *Scale-bars*: a, 500 μm; b, 10 μm; inset 5 μm

Both sexes have elongated, cylindrical and slender bodies, slightly tapered at both, anterior and posterior ends (Fig. 2b). The cuticle at the anterior extremity is smooth, more or less dilated, appearing as a small cephalic vesicle (Fig. 3a). Mouth opening, in both sexes, is terminally placed, slightly triangular, being surrounded by six labial papillae each of them having a small protuberance at the top. At the level of the amphids, there are four cephalic papillae. A single rudimentary triangular tooth is visible in the buccal cavity and a small cutting plate in opposite position. Two amphidial pores are present at the anterior extremity (Fig. 3b). The buccal cavity leads into an oesophagus composed of a cylindrical corpus, slightly widened at its posterior part where it forms a bulb (Fig. 3a).

**Fig. 4:**
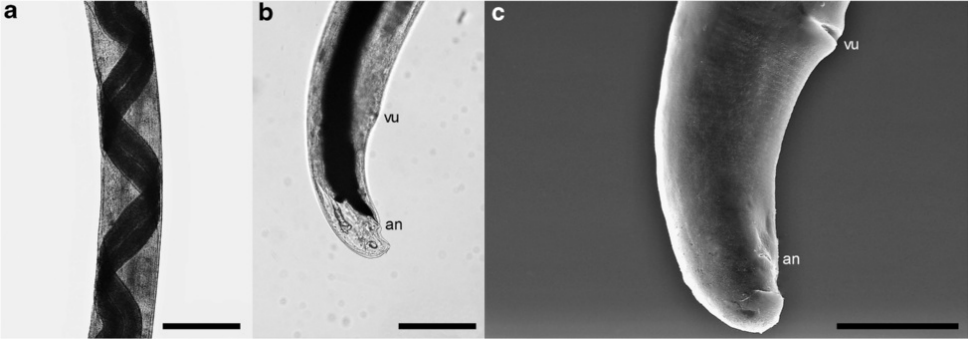
Light microscopy (**a**, **b**) and SEM photomicrographs (**c**) of *A. daskalovi* female. **a** “Barber-pole” appearance of the body. **b** Slightly curved posterior extremity. **c** Ventral view of the posterior extremity showing the vulva (vu) and the anus (an). *Scale-bars*: a, 500 μm; b, 500 μm; c, 100 μm

The females have a “barber pole” appearance due to their discolored uterus coiled with the brownish intestinal tract (Fig. 4a), and a slightly curved posterior extremity (Fig. 4b) showing the vulvar and anal openings on the lower curvature (Fig. 4c). The vulvar opening appears as a transverse slit (Fig. 4c). The anus is oval, transversely elongated and smaller than the vulva.

**Fig. 5:**
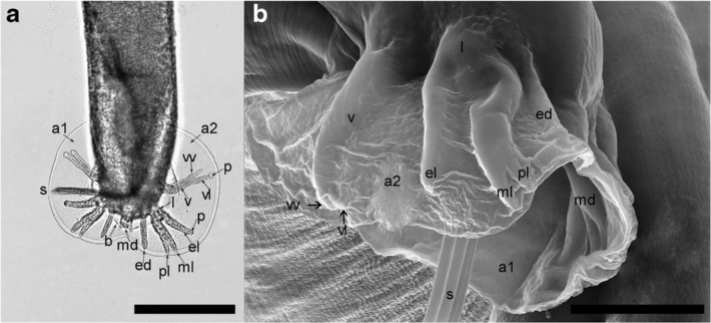
Light microscopy (**a**) and SEM photomicrographs (**b**) of copulatory bursa of *A. daskalovi*: left ventro-lateral lobe (a1); right ventro-lateral lobe (a2); dorsal lobe (b); ventral ray (v); ventro-ventral branch of ventral ray (vv); ventro-lateral branch of ventral ray (vl); protuberances (p); lateral ray (l); externo-lateral part of lateral ray (el); medio-lateral branch of lateral ray (ml); postero-lateral branch of lateral ray (pl); externo-dorsal ray (ed); median dorsal ray (md); spicules (s). *Scale-bars*: a, 200 μm; b, 50 μm

**Fig. 6:**
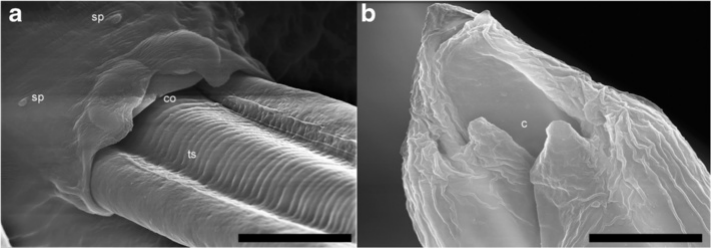
SEM micrograph of adult male of *A. daskalovi*. **a** Details of the cloacal opening (co) with two papillae (papillae 7) (p), sensory papillae (sp) and transverse striations (ts) of the spicules. **b** Detail of the adjoining arrangement of spicules forming a channel (c). *Scale-bars*: a, 10 μm; b, 5 μm

The males had a uniformly coloured body, a small bilobated copulatory bursa, and two unequal spicules. The copulatory bursa had two symmetrical, transparent, ventro-lateral lobes (Fig. 5), the latter supported by rays with a variable layout and different origin: ventral, lateral, externo-dorsal and median lateral. The ventral ray is distally divided into two branches, ventro-ventral and ventro-lateral, the former being slightly shorter than the latter; ventro-lateral branch ends with a protuberance at the top. The lateral ray is split into three branches: externo-lateral, showing a protuberance at the top, medio-lateral and posterio-lateral, the last two being thinner and separated towards the terminal end. The externo-dorsal ray is straight, undivided, and smaller than the previous two. The median dorsal ray is short, thick, strong and rectangular and has two digitations. The spicules are slender, thin, brownish and unequal, with striated alae. They protrude through the cloacal opening, two papillae (papillae 7) being present behind this orifice; several papillary structures, with a presumptive sensorial role, surround the cloacal opening (Fig. 6a). The spicules show transverse striations and the distal ends are corrugated. When the spicules are joined, they form a channel probably used for semen disposal during fertilization (Fig. 6b).

**Fig. 7:**
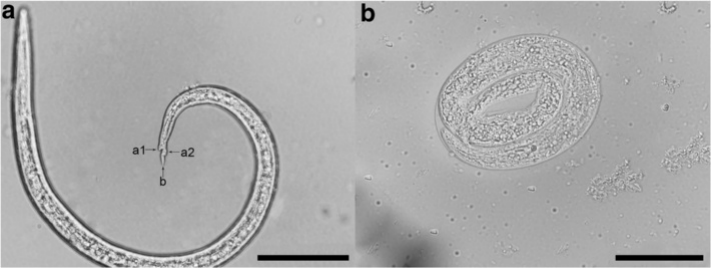
Light microscopy photomicrographs of *A. daskalovi.* L1 larva. **a** Posterior extremity of L1 larva: dorsal spine (a1); subterminal notch (a2); wavy tail (b). **b** Egg. *Scale-bars*: a, 50 μm; b, 50 μm

The measurements of eggs (mean 101 × 79 μm) and larvae (mean 379 × 17 μm) isolated from the lung tissue and faeces are given in Table 2. Stage-two larvae possess the typical metastrongyloid posterior end with a wavy tail, one dorsal spine, and a subterminal notch (Fig. 7a).

**Table 2:** Morphometric data for the eggs and first-stage larvae (L1) of *A. daskalovi*

Species	Stage	Range (μm)		Reference
		Length	Width	
*A. daskalovi*	Eggs (*n* = 10)^a^	98–105	74–90	Present study
	L1 (*n* = 150)	336–412	14–20	Present study
*A. chabaudi*	L1	307–420	15–17	[25]
	L1	362–400	15–18	[31]
*A. vasorum*	L1	310–399	14–16	[24, 32]

^a^Eggs measured in feces. No comparative data for eggs are provided, as the existing data in the literature for *A. chabaudi* and *A. vasorum* refer to measurements of eggs *in utero*

Microscopic examination of the Baermann sediment also revealed the presence of some eggs of *A. daskalovi*. These were embryonated, oval, with thin shells (Table 2, Fig. 7b).

### Pathology

**Fig. 8:**
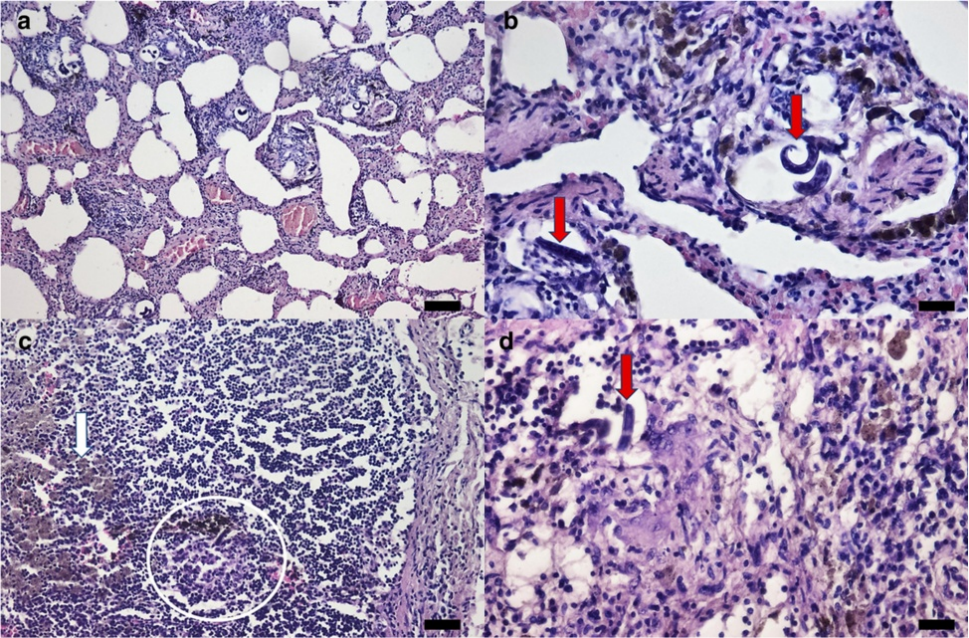
Histological sections (haematoxylin-eosin staining) of the lung and tracheobronchial lymph nodes of badgers infected with *A. daskalovi*. **a** Diffuse hyperemia, fibrosis and granulomatous reaction of the pulmonary interstitium. **b** Coiled larvae in the bronchial tree and interstitium (*red arrows*). **c** Hemosiderin deposits (*white arrow*) and granulomatous reaction surrounding fragments of parasites (*white circle*) in the tracheobronchial lymph node. **d** Detail of granulomatous reaction centered on larvae (*red arrow*) in the tracheobronchial lymph node. *Scale-bars*: a, 100 μm; b, 20 μm; c, 50 μm; d, 20 μm

At necropsy, numerous adult worms were present in the pulmonary arteries and the right atrium, without visible gross morphological changes. The lungs showed diffuse congestion and contained several, variably-sized, firm, gray-red and slightly raised nodules that were randomly distributed within all lung lobes. The tracheobronchial lymph nodes were markedly and diffusely enlarged up to 2–3 times the normal size, irregular in shape, and gray to red on the cross section. The histological findings were consistent with chronic interstitial verminous pneumonia. The fibrous tissue and granulomatous reaction consisting of reactive macrophages, multinucleated giant cells, and fewer neutrophils, eosinophils and plasma cells, occasionally surround the larvae (Fig. 8a).

Larvae were composed of numerous round, basophilic nuclei with scant eosinophilic cytoplasm and a thin amphophilic cuticle (Fig. 8b). Scattered hemorrhagic areas associated with hemosiderin deposits, hemosiderin-laden macrophages (Fig. 8b), and coagulative necrosis were found in the affected parenchyma.

Less affected areas of the lung presented mild congestion, edema, pulmonary atelectasis and alveolar emphysema. The pulmonary arteries exhibited moderate smooth muscle hypertrophy and hyperplasia of the arterial tunica media with mild vacuolar degeneration of the endothelial cells and intimal fibrosis. Multifocal and mild subendocardial fibrosis and minimal mononuclear infiltrates were observed in the right atrium.

The tracheobronchial lymph nodes showed diffuse reactive hyperplasia, multifocal granulomatous reaction centered on parasitic organisms and numerous aggregates of hemosiderin-laden macrophages (Fig. 8c, d). There were no microscopic lesions in the area of pulmonary arteries where adult parasites were located.

### Molecular analysis

**Fig. 9:**
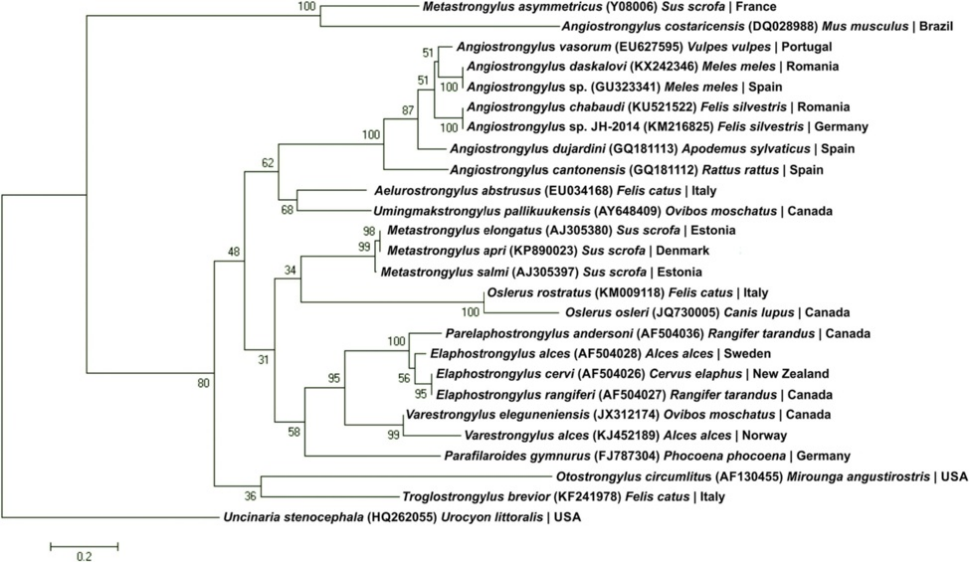
Phylogenetic tree based on ITS2 sequences for different European species of *Angiostrongylus* retrieved from GenBank. The evolutionary history was inferred by using the Maximum Likelihood method based on the Tamura-Nei model. The tree with the highest log likelihood (-9756.7630) is shown. The percentage of trees in which the associated taxa clustered together is shown next to the branches. Initial tree(s) for the heuristic search were obtained by applying the Neighbor-Joining method to a matrix of pairwise distances estimated using the Maximum Composite Likelihood (MCL) approach. The tree is drawn to scale, with branch lengths measured in the number of substitutions per site

All sequences (*n* = 6) obtained from adults and larvae (GenBank accession number KX242346) were identical and showed a 100 % homology to an *Angiostrongylus* sp. recovered from a badger from Spain (accession number GU323341). Phylogenetic analysis clustered *A. daskalovi* within the clade including all other European species *Angiostrongylus* with sequences available in the GenBank database (Fig. 9).

## Discussion

This report represents the first comprehensive study of *A. daskalovi* infection in European badgers. The study identified this species based on morphological, morphometric and molecular analyses. Three species of *Angiostrongylus* are described in different badger species across the world, namely *A. vasorum*, *A. daskalovi* and *A. gubernaculatus*. Morphometrically, *A. vasorum* is the smallest species, the length of females ranging between 14.17–17.69 mm and that of males between 11.21–13.91 mm [23]. These values overlap the lower limits of the ranges for length of *A. daskalovi*: 14.39–31.12 mm in females and 13.36–21.31 mm in males [7]. Our data correspond to specific dimensions of *A. daskalovi*, and may differentiate these two species even if the recorded variations could be related to the intervals after infection [24]. The third species, *A. gubernaculatus* is morphometrically very similar to *A. daskalovi*, but host specificity and geographical range differ amongst the two species. Additionally, another congeneric species, *A. chabaudi* was recently found and redescribed in wildcats in the same geographical area from Romania, providing a detailed comparison of the morphometric features [25].

Scanning electron microscopy revealed similar structures of the anterior and posterior ends as described in adult *A. vasorum* [23], but also identified particular structures. At the anterior extremity, the mouth opening is surrounded by six papillae and two amphidal pores, but the small tooth observed in the mouth cavity of *A. daskalovi* is not known in *A. vasorum*. Comparison of the copulatory bursa of *A. vasorum* with that of *A. daskalovi* reveals only a slight difference in the median dorsal ray whose digitations are sometimes separated by a papilla, but none of the males of *A. daskalovi* exhibited this structure.

Despite the morphometric differences, these three species may have a common origin. *Angiocaulus gubernaculatus* which resembles *Angiostrongylus* sp., currently accepted as a synonym [26], may represent a common ancestor for the Brazilian and European populations of *A. vasorum*. Some authors consider that *A. vasorum* is the ancestral species that subsequently spread globally with its carnivore hosts, and evolved into genetically distinct populations in various host species [27]. The phylogenetic analysis performed herein is also supportive of this hypothesis, as *A. daskalovi* clustered with *A. vasorum*.

To our knowledge, this is the first description of the first-stage larva (L1) of *A. daskalovi*. The differentiation of first-stage larvae of different *Angiostrongylus* species is difficult due to several common characters such as the transparent body, sigmoid tail, the presence of a dorsal spine and a visible notch. Their lengths partly overlap from one species to another: 310–400 μm for *A. vasorum* and 336–412 μm for *A. daskalovi* in the present study (L1s of *A. gubernaculatus* are not described). This does not allow a clear differentiation based on morphological and morphometric criteria. However, the adults and larvae of *A. daskalovi* identified in this study were 100 % similar to adults of *Angiostrongylus* sp. recovered from a badger in Spain and tentatively identified, based on morphology and morphometry, as *A. daskalovi* [8].

The presence of molting larvae in tracheobronchial lymph nodes and eggs in the lung parenchyma provides evidence that the life-cycle of *A. daskalovi* is probably similar to that of *A. vasorum*, following the type II of the known development in the definitive hosts [28]. During their migration, larvae may exert a significant pathogenic action. Hypertrophy and hyperplasia of the arterial tunica media are explainable by pulmonary hypertension due to the presence of nematodes in the lung arteries, being similar to those lesions recorded in dogs naturally infected with *A. vasorum* [29]. The presence of hemosiderin deposits and hemosiderin-laden macrophages, edema, pulmonary atelectasis, alveolar emphysema, diffuse congestion of the lungs and the presence of the nodules randomly distributed within all lung lobes are similar to those produced by *A. vasorum* in dogs [30]. All these pathological alterations of the lungs and the pulmonary arteries confirm that *A. daskalovi* might play an important pathogenic role in infected badgers.

Although the species of *Angiostrongylus* infecting carnivores seem to show a relatively well-defined host specificity, some studies report certain overlaps. As badgers can occasionally be infected with *A. vasorum*, dogs might also be infected occasionally with *A. daskalovi*. Our new molecular data and larval morphology can partly solve possible misdiagnosis problems in European carnivores. Still to be solved is the life-cycle of *A. daskalovi*.

## Conclusions

The current study confirms the existence of *A. daskalovi* patent infection in badgers from Romania and provides the first description of the larvae, its pathological effect, and its phylogenetic relationships with other congeneric species.

## Abbreviations

BLAST, Basic local alignment search tool analysis; DNA, deoxyribonucleic acid; H&E, Hematoxylin and eosin; L1, First larval stage; PCR, polymerase chain reaction; SEM, Scanning electron microscopy
